# Functional Allele Validation by Gene Editing to Leverage the Wealth of Genetic Resources for Crop Improvement

**DOI:** 10.3390/ijms23126565

**Published:** 2022-06-12

**Authors:** Michael J. Thomson, Sudip Biswas, Nikolaos Tsakirpaloglou, Endang M. Septiningsih

**Affiliations:** Department of Soil and Crop Sciences, Texas A&M University, College Station, TX 77843, USA; m.thomson@tamu.edu (M.J.T.); sudipbmb@tamu.edu (S.B.); n.tsakirpaloglou@ag.tamu.edu (N.T.)

**Keywords:** CRISPR-Cas9, genome editing, crop biotechnology, genomics, candidate genes, causal variants, allele mining

## Abstract

Advances in molecular technologies over the past few decades, such as high-throughput DNA marker genotyping, have provided more powerful plant breeding approaches, including marker-assisted selection and genomic selection. At the same time, massive investments in plant genetics and genomics, led by whole genome sequencing, have led to greater knowledge of genes and genetic pathways across plant genomes. However, there remains a gap between approaches focused on forward genetics, which start with a phenotype to map a mutant locus or QTL with the goal of cloning the causal gene, and approaches using reverse genetics, which start with large-scale sequence data and work back to the gene function. The recent establishment of efficient CRISPR-Cas-based gene editing promises to bridge this gap and provide a rapid method to functionally validate genes and alleles identified through studies of natural variation. CRISPR-Cas techniques can be used to knock out single or multiple genes, precisely modify genes through base and prime editing, and replace alleles. Moreover, technologies such as protoplast isolation, in planta transformation, and the use of developmental regulatory genes promise to enable high-throughput gene editing to accelerate crop improvement.

## 1. Introduction

Since the advent of molecular markers, key genetic loci controlling traits of interest have been mapped in major crop plants. Using natural variation and mapping populations, thousands of major genes and quantitative trait loci (QTLs) have been mapped to chromosomal regions, but few causal genes have been identified through map-based cloning. At the same time, whole genome sequencing has led to high-quality reference genomes. Comprehensive sets of annotated genes and functional genomics efforts have attempted to assign gene function through sequence similarity analysis, large-scale mutant populations, and whole genome expression profiling. More recently, next-generation sequencing has led to high-resolution single nucleotide polymorphism (SNP) haplotype maps and the identification of putative causal variants across large resequencing studies. Taken together, these resources have prepared the foundation for a new era of functional validation of genes and alleles for crop improvement using CRISPR-Cas technologies. This review will explore the current state of the art in crop genetics, genomics, and gene editing that promise to enable high-throughput genome editing for more powerful crop improvement strategies for the future.

## 2. Integrating Genetic Mapping and Genomics for Candidate Gene Identification

**Figure 1:**
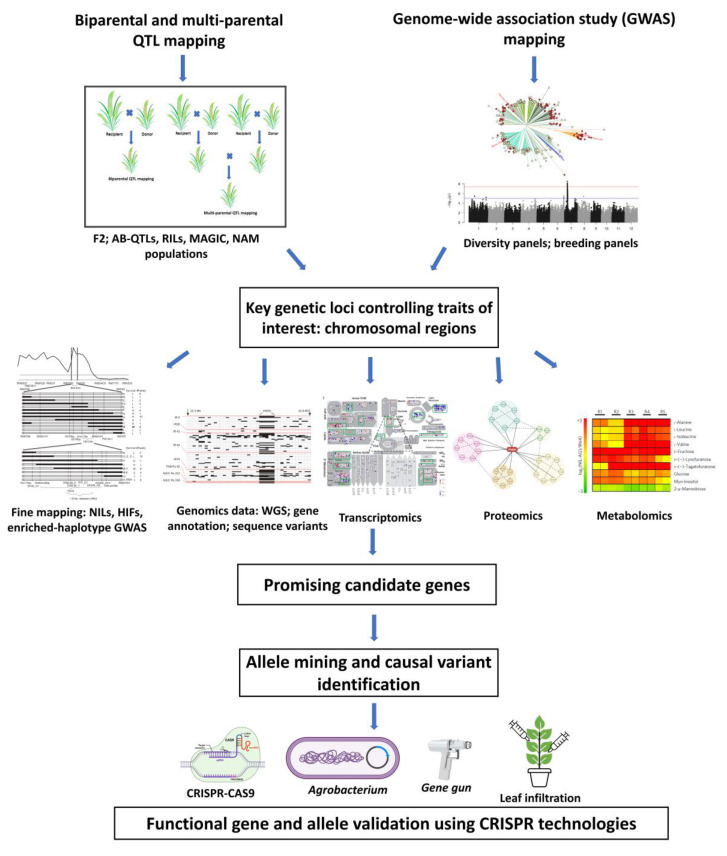
Scheme for integrating genetic mapping and genomics data to identify candidate genes and causal variants, which can then be validated using CRISPR technology (partially created with BioRender.com, accessed on 23 March 2022).

Genetic linkage mapping studies and genome-wide association studies (GWAS) have identified a wealth of genes, QTLs, and QTL-GWAS regions for various traits over the last few decades. Many of these data are available in public databases, such as the GrainGenes database for wheat and oat (https://wheat.pw.usda.gov/GG3/, accessed on 23 March 2022), MaizeGDB for maize (https://maizegdb.org/, accessed on 23 March 2022), and QTARO ([1]; http://qtaro.abr.affrc.go.jp/, accessed on 23 March 2022) and Gramene (https://www.gramene.org, accessed on 23 March 2022) for rice. As mapped QTLs provide a direct link to breeding-relevant genetic loci, new strategies need to be explored to integrate genetic mapping, genomics, and precision phenotyping to identify candidate genes underlying key QTLs of interest (Figure 1). These candidate genes can then empower more advanced breeding techniques, including CRISPR-Cas gene editing. Moreover, the easing of the biotechnology regulatory process for gene-edited crops has attracted the interest of scientists to dig deeper beyond QTL regions to accelerate crop improvement using the latest gene editing technology.

### 2.1. Identifying Chromosomal Regions Controlling Traits of Interest

With the availability of low-density genetic markers, beginning with restriction fragment length polymorphism (RFLP) markers and later with simple sequence repeat (SSR) markers, the first efforts to statistically measure the contributions of chromosomal regions to a trait of interest were performed using biparental linkage mapping populations [2]. Subsequently, more complex multiparental linkage mapping approaches emerged alongside higher resolution genetic markers. Likewise, the availability of higher density genetic markers also opened the era of genome-wide association studies. There are advantages and disadvantages to each of these strategies [3,4].

#### 2.1.1. Linkage Mapping Populations

Various options for biparental mapping populations are available, depending on the objective of the study, the trait of interest, species, and availability of time and funding. Although this strategy suffers from limited genetic diversity from employing just two parents, this drawback may be complemented by other genetic techniques in a later stage, including allele mining [5]. Some examples of biparental mapping include backcross, F_2_, and recombinant inbred line (RIL) populations. The advanced backcross QTL (AB-QTL) strategy is a method to simultaneously identify QTLs and transfer beneficial QTLs from unadapted germplasm into elite lines for crop improvement to tap the genetic potential from the wild relatives [6,7]. This strategy uses an exotic genetic donor with several backcrosses to cultivated germplasm to better identify trait-enhancing alleles, and has been used extensively in tomato [8,9] and rice [10,11,12]. Another approach is to use an F_2_ population, which requires individual plants to be genotyped and phenotyped. An alternative is to use the F_2:3_ generation for more accurate phenotyping, although there is some QTL detection power lost due to segregation within families. Our work in rice for flooding-tolerant traits, for example, employed F_2:3_ populations with good success [13,14,15,16]. Such populations can be generated in a short time; however, they have limited use due to constraints in the number of available seeds. To overcome the seed constraint issue, an immortalized mapping population, such as a doubled haploid (DH) or recombinant inbred line (RIL) population, can be used to provide fixed homozygous lines. RIL populations are developed using single seed descent from F_2_ individuals, which can be sped up if a rapid generation advance (RGA) facility is available [17]. The advantage of using DH or RIL populations is that the population only needs to be genotyped once, but unlimited replicates are available for more accurate phenotyping of one or more traits. RIL populations have been used in our research to efficiently map various abiotic-stress-tolerance traits in rice [18,19,20] as well as for various traits in peanuts [21,22,23].

More complex mapping populations include the multiparent advanced generation intercross (MAGIC) and the nested association mapping (NAM) populations. A MAGIC population involves the use of multiple founders with one or more cycles of intercrossing followed by selfing. Similar to RILs, this population can be genotyped once with unlimited replicates available; however, MAGIC offers higher genetic diversity and finer mapping resolution over biparental DH and RIL populations. An excellent example in rice is the set of MAGIC populations developed by the International Rice Research Institute [24]. These populations serve as permanent mapping populations to precisely identify beneficial loci as well as to provide superior lines for varietal development. The NAM populations were first introduced in an outcrossing species, maize [25], by crossing multiple inbred lines to a single reference line and then developing multiple biparental mapping subpopulations. This type of population offers a high mapping resolution across a diverse set of founders, providing the ability to precisely map a wide range of QTLs associated with complex traits. To develop such a complex population, however, requires significant time and effort. The first NAM population developed involving 25 maize inbred lines crossed to a single recurrent parent, resulting in 200 RILs per sub-population, totaling 5000 RILs [26]. This NAM resource has been widely used by the maize community and has led to the identification of thousands of QTLs as well as several causal genes [27].

#### 2.1.2. Genome Wide Association Studies (GWAS)

The first GWAS in plants was performed in *Arabidopsis* [28] and shortly thereafter in maize [29]. GWAS involves genotyping and phenotyping of many individuals in a diversity panel or breeding population for the traits of interest and statistically testing the association of the chromosomal regions with the phenotype. Generally, only more common alleles can be detected, but with a much finer mapping resolution than with linkage mapping populations. Genotyping in GWAS can be performed using SNP chip arrays at different resolutions, or more recently, genotyping by sequencing approaches. For example, GWAS in rice has been performed using a 7K array [30,31,32], 44K array [33], 600K array [34], or genotyping by sequencing [35]. With more affordable next-generation sequencing, larger populations of crop germplasm accessions can be sequenced for more powerful GWAS, for example, the use of the 3000 Rice Genomes project, which was sequenced with 14X coverage [36]. Numerous GWAS have been performed after the sequencing data and the associated rice seed materials became available (for example, [37,38]). Additionally, with the ease of high throughput phenotyping, GWAS has become a more attractive tool to dissect the molecular genetic control of breeding-relevant traits, for example, in rice [39], sorghum [40], and corn [41].

### 2.2. Identifying Promising Candidate Genes

Identifying candidate genes underlying the QTL of interest is an essential next step for further functional validation and confirmation of the causal gene(s) and related pathway. Once identified, the causal genes can be targeted for modification using genetic tools, such as gene editing. Promising candidate genes can be sought with one or combinations of the strategies described below, depending on the complexity of the QTL.

#### 2.2.1. Fine Mapping

Fine mapping is needed when the QTL region is too large. Fine mapping will help in narrowing down the region to fewer candidate genes and also assist in molecular breeding to reduce the negative linkage drag, especially when the donor is a non-elite variety (including traditional landraces or wild relatives). The first step for fine-mapping is to develop near-isogenic lines (NILs) to isolate the QTL effect in an otherwise identical genetic background. For example, NILs targeting the *SUB1* region for tolerance to submergence during the vegetative stage were developed in the background of a number of popular but susceptible varieties, with selection for flanking recombinants to reduce the negative linkage drag surrounding the *SUB1* locus [42,43,44]. NILs are useful to increase the power of detecting small-effect QTLs, as well as to provide a platform for further fine-mapping by identifying new recombination breakpoints flanking the QTL. Some examples in rice include the fine mapping of QTLs for flowering time [45], pericarp color [46], and anaerobic germination [47]. An excellent example of detecting recombinants within the candidate gene of the QTL was demonstrated by the fine mapping of a plant height QTL, *ph1.1*, detected on chromosome 1 in an AB-QTL (BC_2_F_2_) population derived from IR64 and *O. Rufipogon.* This QTL was successfully fine-mapped within two recombination breakpoints of a 2157 bp interval within the GA 20-oxidase candidate gene. This was achieved by generating 1300 BC_2_F_3_ individuals, where 13 recombinants were subsequently identified, including two within the candidate gene region. Upon sequencing the gene, the IR64 allele differed from O. *Rufipogon* by a 382 bp deletion, which deleted parts of the first and second exons and caused a frameshift, creating a novel termination codon, effectively knocking out the gene and resulting in the semi-dwarf phenotype [10,48,49]. The cloning of this gene, which turned out to be the green revolution gene *sd1*, was simultaneously reported by multiple research groups [50,51,52,53].

To circumvent the length of time in developing NILs, heterogeneous inbred families (HIFs) can be used as an alternative. HIFs take advantage of a single introgression segment in heterogeneous backgrounds. Other than increasing the power to detect small-effect QTLs, these lines also increased the power to detect epistasis [54,55]. An example of this work in rice is the use of HIFs for confirming the relative contribution of three QTLs controlling blast resistance disease derived from an SHZ-2 to TXZ-13 cross [56]. Another alternative to NILs is the use of enriched-haplotype GWAS. Using data from the 3000 Rice Genomes Project, a diversity panel based on the genetic donor (Ma-Zhan Red) haplotype in the target region for the *AG2* QTL for anaerobic germination located on chromosome 7 was used in a GWAS to successfully narrow down the region of the QTL from more than 7 Mb to less than 0.7 Mb [38]. 

#### 2.2.2. Integrating Various Types of Genomics Data

Whole genomic sequence (WGS) data, gene annotations, transcriptome, proteome, and metabolome data are increasingly becoming more widely available. These data can be used to support the identification of potential candidate gene(s) underlying the QTL of interest (Figure 1). For example, a study in rice used WGS to provide a more detailed profile of a QTL region for submergence tolerance, *qSub8.1* [57]. Another example combined enriched-GWAS QTL and transcriptomic data to identify promising candidate genes for an anaerobic germination QTL, *AG2* [38]. Lastly, [47] combined fine mapping with transcriptomics and metabolomics to pinpoint the most promising candidate gene underlying another QTL for anaerobic germination, *AG1*. Many other examples are available in recent reviews, for example, in tomato [58], soybean [59], and maize [60]. 

## 3. Allele Mining and Causal Variant Identification

Although integration of genomics data with high-resolution QTL mapping has accelerated the process of identifying candidate genes, there remains a bottleneck in narrowing down the number of potential candidate genes at each target locus. This bottleneck is caused by several reasons. First, increasing the resolution of biparental linkage mapping requires developing very large fine-mapping populations that can be difficult and expensive to implement. For example, a review of 41 positional cloning studies in rice showed a median fine-map population size of 2419 genotyped progeny was needed to map to an average of 44.5 kb resolution, narrowing down the target region to approximately five genes [61]. Secondly, a best-case GWAS experiment using high-resolution SNP mapping and a sizable diversity panel can narrow a QTL to roughly the level of linkage disequilibrium (LD) in the target germplasm, which in rice averages 100 kb across diverse sets of *Indica* germplasm and can exceed 200 kb in more narrow germplasm pools, such as *Tropical* or *Temperate japonica* [62]. One approach to provide even greater mapping resolution is to leverage the power of linkage mapping and GWAS in the same experiment. For example, a recent study performed joint linkage mapping and GWAS for the level of carotenoids in maize, and 11 out of 44 detected QTLs were resolved to individual genes, of which six had strong correlations between gene expression and the QTL allele effect explained by those loci, providing useful targets for improving carotenoid traits in maize [63]. 

Once GWAS and linkage mapping approaches have been exhausted, there will likely still be multiple annotated genes in the target region. As mentioned above, genomics data, such as predicted function based on sequence homology and gene expression data, can help prioritize the list of candidate genes, but is rarely enough to definitively identify the causal gene underlying the QTL. Another layer of information is needed to add evidence as to which candidate is the causative gene: in this case, next-generation sequencing (NGS) data can provide a valuable source of data on haplotypes, alleles, and causal variants, as described below. This rapidly expanding set of NGS data promises to usher in a new era of what has been referred to as “genomics-assisted breeding v2.0” that integrates data from genome-wide mapping, genomics, and haplotypes to identify causative genes and design future crops [64].

### 3.1. Allele Mining Using Whole Genome Sequence Data

The advent of high-throughput DNA sequencing for the Human Genome Project, initially with capillary electrophoresis systems and followed by next-generation sequence techniques, quickly expanded from sequencing a single genome to characterizing the suite of DNA variants at the population level [65]. These efforts led to the large-scale identification of single nucleotide polymorphism (SNP) markers, which provided a higher resolution than previous marker systems [66]. The information content of SNPs is increased by considering the pattern of physically linked SNP markers along the chromosome, which is called an SNP haplotype [67]. Although resolving haplotypes of largely heterozygous species can be challenging, for the purposes of this review, an SNP haplotype at a candidate gene locus will be used synonymously to describe the “allele” at that locus, whether in largely homozygous (such as rice) or heterozygous species (such as maize). Once sequence variants are characterized at the population level, then linkage disequilibrium (LD) can be defined. LD is the non-random association of alleles at different loci generally due to physically linkages from being inherited from a common ancestor, which is demonstrated as SNP haplotype “blocks” across the genome and forms the basis for genome-wide association studies (GWAS).

The idea of using DNA sequence data to move beyond mere association to identify the genetic polymorphisms contributing to a phenotype has been explored since large-scale sequencing began being implemented in the 1990s. In human and model genetic systems, the term “causal variant” was used to describe the specific genetic polymorphism that led to the observed phenotypic difference, either in humans or model systems such as mice [68]. Although initial efforts to describe causative mutations dealt with simple traits, approaches were quickly implemented to apply towards complex traits, with causative variants underlying QTLs also being referred to as “quantitative trait nucleotides” (QTN) [69]. The term “functional nucleotide polymorphism” (FNP) began being used to describe causal variants in plants [70]. An important concept was raised at the time: were common human diseases caused by common variants? If there is substantial allelic heterogeneity at a target locus across a population, it will be more difficult to identify the causative allele and gene since different alleles at a locus may be contributing to the same phenotype [71]. This has led to modern efforts to move beyond SNP–trait associations and instead use next-generation sequence data to characterize the spectrum and frequency of alleles at each locus towards identifying putative causal variants [72].

Early efforts to describe “genome to phenome” relationships in human genetic research [73] have become a central theme in plants, as researchers work to identify which genetic variants lead to specific phenotypes. Three model plants have led the way in large-scale next-generation sequencing, SNP haplotype characterization, and identification of causal variants: *Arabidopsis*, rice, and maize. In *Arabidopsis*, one of the goals of the 1001 Genomes Project is to identify functional variants; notably, an initial study of 80 sequenced *Arabidopsis* genomes found 2793 genes with premature stop codons in two or more accessions [74]. Likewise, a study of 19 *Arabidopsis* genomes combined with RNA-Seq data was able to identify potential cis-acting functional variants associated with differential gene expression, providing evidence for causal variants located in the promoter regions [75].

In rice, the 3000 Rice Genomes Project performed whole genome sequencing of 3010 rice accessions and identified 17 million SNPs and 93,683 structural variations, including insertions, deletions, and translocations [36]. This dataset can be queried using the Rice SNP-Seek database, which shows SNPs and short insertion/deletion sites across the 3000 accessions for any specific genetic locus [76]. This dataset has subsequently been used for precise GWAS experiments down to the gene level, such as a study for salinity tolerance using a subset of 664 sequenced rice accessions that identified two major loci, along with putative candidate genes and causal variants at each locus [37]. Allele mining using the 3000 rice genomes was also performed to identify beneficial alleles for disease resistance genes in rice [5]. An online tool to help mine beneficial haplotypes from the 3000 genomes data is the Rice Functional and Genomic Breeding (RFGB) v2.0 resource that allows a user to start with a phenotype to identify haplotype associations and the corresponding candidate genes [77]. Another large-scale study of potential causal variants across the rice genome was recently conducted by whole genome sequencing of 4726 rice accessions, leading to the identification of over 17 million total variants, of which were 918,848 missense mutations in coding sequences; when combined with chromatin accessibility data, this dataset provides a resource to prioritize possible causal variants underlying any genetic locus [78].

Lastly, large-scale efforts for resequencing and SNP haplotype characterization in maize have provided rich datasets for allele mining and causal variant identification. The HapMap2 project used a population genetics-based model to identify 55 million SNPs in 103 lines across *Zea mays* varieties, including potentially functional variants, with 1500 of the high confidence genes having premature stop codon variation [79]. An expanded HapMap 3 initiative then used whole genome sequence data from 1218 maize accessions and lines to identify over 83 million variant sites [80]. These datasets have been supplemented with large-scale transcriptome analysis, such as a study of 255 lines across seven tissues that mapped eQTLs and identified rare deleterious variants across the maize genome [81]. These efforts across *Arabidopsis*, rice, and maize have set the foundation for leveraging next-generation sequence data to identify causal variants controlling key traits of interest.

### 3.2. Categories of Causal Variants

Now that large-scale deep sequence data are widely available for major crop species, candidate polymorphisms that may represent causal variants for different traits can be cataloged and prioritized for further study. An important concept is that any accession, or even an individual plant, has a suite of thousands of mutations in their genome, each with different histories and potential for functional impact. One of the future challenges in the field will be finding approaches to narrow down lists of functional variant candidates to find which ones are the causal variant for any specific gene and trait. The first category of possible functional variants is those that affect protein structure. These include obvious mutations that knock out gene function, such as gene deletions, transposon insertions, or premature stop codons that lead to truncated proteins. More subtle differences in protein structure can result from alternative splicing leading to protein isoforms, as well as amino acid substitutions, or small insertion/deletions in exons, leading to altered protein structures. The next category of functional variants is those affecting gene expression. These include variation in cis-regulatory elements in the promoter regions, as well as long-range cis-regulatory elements, such as those characterized in the maize genome [82]. Another category of functional variants includes epigenetic variants, such as DNA methylation and histone post-translational modifications, which can lead to epialleles that can cause heritable phenotypic differences without changes in the underlying DNA sequence [83].

### 3.3. The Value in Identifying Causal Variants in the Age of CRISPR/Cas Editing

The wealth of genomics data enables identification of candidate genes and potential functional variants underlying a target locus, but a major bottleneck remains the process of proving the specific causal variants contributing to the phenotype of interest. The advent of efficient CRISPR/Cas-based gene editing now provides an approach to validate specific polymorphisms and their effect on phenotypes (described in detail below), as well as deploy beneficial polymorphisms for crop improvement. Whereas previous approaches, such as marker-assisted selection and genomic selection, did not require knowledge of the causal gene and allele, future molecular breeding strategies will be able to target functional allele modifications to accelerate genetic gains and enable novel trait development. The value in identifying causal variants in the age of CRISPR/Cas editing has been proposed as a new technological phase called “Breeding 4” that leverages massively parallel gene editing to directly select at the level of causal variants [84]. In addition to high-throughput gene editing, additional enabling technologies for Breeding 4 include trait decomposition based on physiological trait components, the selection of endophenotypes, or “intermediate molecular traits such as gene expression levels or metabolic activity,” and inferring traits directly from precision phenotyping image data, which can be analyzed through convolutional neural networks [84]. Related to this vision, a holistic view of crop improvement has been proposed that combines the latest technologies for genome assembly, germplasm characterization, gene identification, genomic-assisted breeding, and gene editing, in combination with a rapid cycle breeding strategy [85]. Moreover, targeting causal genes and alleles can enable non-transgenic gene editing approaches that fall under USDA SECURE rule exemptions and, therefore, are more widely accessible to public breeding programs that would not previously have been able to afford the transgenic deregulation process.

## 4. Functional Gene and Allele Validation Approaches

### 4.1. Gene Knockouts

#### 4.1.1. Single Gene Knockouts

Plant genome editing occurs by utilization of programmable sequence-specific nucleases (SSNs) that generally include engineered endonucleases or meganucleases, zinc-finger nucleases (ZFNs), transcription activator-like effector nucleases (TALENs), and the CRISPR-Cas system [86]. The common characteristic of these SSNs is the induction of double-strand breaks (DSBs) at target sites, followed by precise genome modifications via DNA repair pathways, such as the non-homologous end joining (NHEJ) or the homology-directed repair (HDR) [87]. In the case of meganucleases, ZNFs, and TALENs, recognition of the target sequences occurs through protein–DNA interactions, whereas the CRISPR-Cas systems rely on the homology of the target DNA sequence and the programmable “guide” RNA, using Watson–Crick base pairing. Evolutionary classification of the CRISPR-Cas systems suggests the existence of two major class systems [88], with the Cas9 nucleases to be included in the second class that mainly utilizes single proteins for inducing double-strand breaking at target sites [89,90]. The simplicity and efficiency of the CRISPR-Cas system has established it as the preferred genome editing method [86,91,92].

To date, two CRISPR-Cas systems are mainly used for plant genome engineering, the Cas9 nuclease and the Cas12a (or Cpf1) nuclease [92,93]. Both Cas variants utilize the NHEJ pathway to induce indel mutations, gene deletions, or gene insertions/replacements, whereas, the HDR pathway has lower frequency and is mainly utilized to provide gene corrections and insertions/replacements when a DNA donor template is provided [92]. The general procedure for plant genome editing is a stepwise process that involves the selection of target sequence, the design and in vitro validation of the sgRNAs, development of genome-editing vectors and their validation in protoplast cells, the in planta delivery of the genome editing components, and the screening and genotyping of the transformed plants for the detection of the targeted edits [86]. Recent examples of single-gene knockouts in rice include knocking out the Sub1 gene in an indica rice cultivar and using a PDS gene knockout to optimize transformation in a tropical japonica rice cultivar [57,94]. To date, two genome-edited products have been commercialized for human consumption, a TALEN-edited soybean that produces oil free of trans fats [95], and a CRISPR-Cas9-edited tomato enriched in GABA [96].

#### 4.1.2. Multiplex Genome Editing

Multiplexed genome editing in plants can be used for regulating the gene expression of various genes, for stacking traits, the control of regulatory pathways, or the removal of deleterious alleles [86,97]. Many convenient and efficient multiplexed sgRNA systems for CRISPR-Cas9 have been developed for the genome editing of plants. In RNA polymerase III (Pol III)-driven systems, Pol III promoters (e.g., U3 and U6) have been employed to regulate the expression of multiple sgRNAs [98,99]. Moreover, utilization of the endogenous RNase P and RNase Z enzymes for processing of the pre-tRNAs transcripts enabled expression of multiple sgRNAs, flanked by tRNA sequences, under the control of a single Pol III promoter [100]. Similar strategies for the simultaneous expression of multiple sgRNAs have been developed using the Pol II promoter-driven systems. In these cases, expression and processing of the poly-sgRNA-containing transcripts could be facilitated by ribozyme sequences flanking the sgRNAs [101], by utilizing polycistronic tRNA-gRNA transcripts inserted into introns [102], or the addition of 6 to 12 bp linkers to flank the sgRNAs [103]. Recently, a more efficient Csy-type ribonuclease 4 (Csy4)-processing system, driven by a Pol II promoter, was demonstrated to enable in planta expression of multiple sgRNAs, separated by specific 20-nucleotide sequences [104]. High-efficiency multiplex gene editing using engineered CRISPR-Cas12a with simple mature direct repeats has also been demonstrated in rice [105].

Most examples of multiplex genome editing in plants involve a single type of editor in combination with multiple sgRNAs that can be used to simultaneously target two (or more) different sites [97]. To date, multiplex gene editing has been mainly utilized to edit homeoalleles and gene families, such as the simultaneous editing of multiple α- and γ-gliadin isoforms in hexaploid bread wheat, resulting in the successful mutation of 35 genes that led to a reduction in immunoreactivity by 85% in a single line [106]. In another example, the multiplex gene editing approach facilitated partial domestication of wild tomato (*Solanum pimpinellifolium*) whilst retaining the stress-tolerance trait of the wild strain [107,108], or enabled the de novo domestication of allotetraploid wild rice genotype *Oryza alta* into a new staple cereal [109]. Multiplex editing also has the potential to simultaneously modify combinations of candidate QTLs or all the genes identified in one QTL region to produce changes to measurable phenotypes [86]. Likewise, multiple genes controlling a single trait can be targeted, such as the editing of four starch branching enzymes in rice [110]. Other multiplexed orthogonal strategies that have been utilized in plants include a catalytically inactive Cas9 (dCas9) with different sgRNA scaffolds (scRNAs) [111], a Cas protein with two sgRNAs (one with a full length protospacer and one with a truncated protospacer) [112], the multiple expression of Cas orthologues [113,114], the simultaneous and wide editing induced by single systems (SWISS) [115], or a combination of the above [116].

### 4.2. Allele Modifications

#### 4.2.1. Base Editing

**Figure 2:**
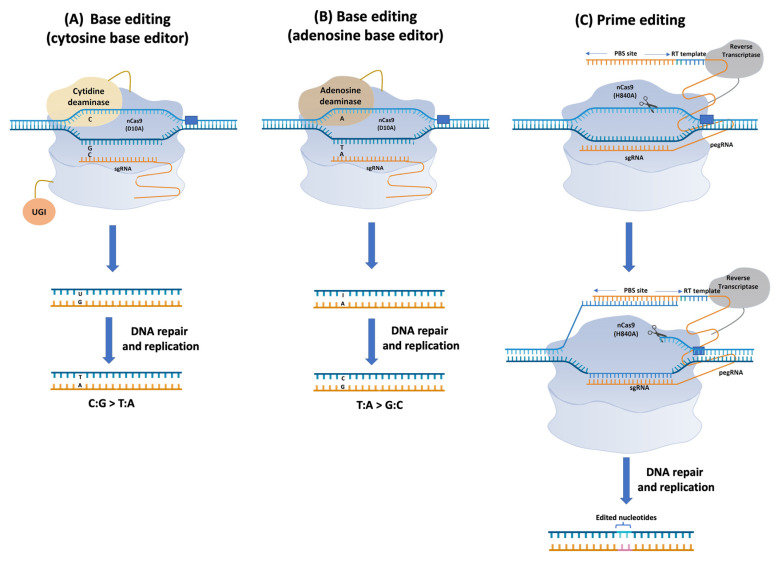
Base editing and prime editing mechanisms in plants are shown: (**A**) cytosine base editors cause C to T (or G to A) conversions; (**B**) adenosine base editors cause T to G (or A to C) conversions, using a dCas9-deaminase fusion protein. While base editors only account for 4 of 12 possible changes, (**C**) prime editing can affect any base substitution or small insertion/deletion, using a Cas9 nickase, reverse-transcriptase enzyme, and a prime editing guide RNA (created with BioRender.com, accessed on 23 March 2022).

Base editing is a recently developed technology that enables the precise modification of genomes at a single-base resolution, without the requirement of DSBs [93,117]. The currently available DNA base editors (BEs) in plants are categorized into cytosine BEs (CBEs), adenine BEs (ABEs), C-to-G BEs (CGBEs), dual-BEs and organellar BEs. The two main classes of BEs, CBEs [118,119,120,121] and ABEs [122] (Figure 2), are fusions of catalytically impaired Cas9 (nCas9 D10A) nucleases with single-stranded DNA (ssDNA)-specific deaminases to facilitate the C:G-to-T:A and A:T-to-G:C base transitions, respectively. In the case of CBEs, cytosine deaminase enables cytosine (C) to uracil (U) conversion in the released ssDNA, and the subsequent repair of the U:G mismatch to U:A that ultimately results in a T:A base pair. Moreover, inclusion of a uracil glycosylase inhibitor (UGI) at CBEs further improves their editing efficiencies by preventing the excision of Us [123]. For ABEs, an *E. coli* tRNA-specific adenine deaminase enables the adenine (A) to inosine (I) conversion, which can be recognized as guanine (G) [122]. To date, CBEs and ABEs have been tested in several plants, including *Arabidopsis* [124,125,126], as well as other dicot species [117]. The activity editing window, as well as the efficiency of these BEs, may vary depending on the target, the GC content, and the respective crop [117]. To improve the efficiency, precision, and specificity of Bes, several approaches have been considered [93] including utilization of engineered deaminases of higher activity or prolonging the exposure of the ssDNA region, altering the spatial location of the deaminase or recruiting multiple copies of the deaminase, reducing indels of CBEs by increasing the copy of the UGI, reducing off-target effects by employing deaminases with lower affinity for ssDNA, and preventing epigenetic modification by utilizing engineered deaminases.

#### 4.2.2. Prime Editing

Prime editing is a recently developed technology that enables all twelve possible transitions and transversions of DNA bases, as well as the predefined DNA insertions and deletions [127]. Prime editors (PEs) are composed of a Cas9 nickase (H840A), which is fused to an engineered reverse-transcriptase (RT) protein, and a prime editing guide RNA (pegRNA) (Figure 2). The pegRNA is a modified sgRNA that contains an RT template encoding the desired edits and a prime binding site (PBS) for hybridization of the 3′ of the nicked DNA strand to initiate reverse transcription. Initially, the Cas9 nickase nicks the non-target DNA of the target site, releasing a ssDNA complementary to the PBS and serves as the primer for the reverse transcription. Launch of the reverse transcription further induces transcription of the RT template that generates a 3′ DNA flap followed by a 5′ DNA flap that subsequently enables incorporation of the desired edit to the target site [127]. In human cell lines, three different PE systems/strategies have been tested (PE1, PE2, and PE3) and it has been demonstrated that the PE2 system that incorporates a wild-type Moloney murine leukemia virus RT (M-MLV-RT) penta-mutant (D200N/L603W/T330P/T306K/W313F) with increased thermostability, processivity, DNA-RNA substrate affinity and inactive RNase H activity exhibited higher editing efficiencies compared to the other two strategies [127].

In plants, all three PE systems/strategies, originally developed for human cells [127], have been tested [117]. Unlike human cell lines, differences in the editing efficiencies between the three PE systems were negligible when tested in different plant species [128,129,130,131]. Moreover, the editing frequency detected in most cases was <2% [117], which is also in contrast with the higher frequencies observed in human cell lines [127]. To optimize the editing efficiency in plants, a surrogate plant prime editor 2 (pPE2) system was tested in rice and led to the generation of T_0_ lines of up to 31.3% frequencies [130]. In a different study based on the PE3 platform, N-terminal fusion of the M-MLV-RTR to Cas9 nickase (H840A) yielded average editing efficiencies up to 24.3% across the thirteen target sites in rice when the pegRNAs contained RT sequences with multiple-nucleotide substitutions [132]. Moreover, testing of the same system in maize protoplasts resulted in an editing efficiency averaging approximately 6% for the four endogenous target sites [132]. In another study, designing of the PBS with a melting temperature of 30 °C, in combination with the in trans expression of the two pegRNAs, resulted in significant improvement in the editing efficiency of PEs in rice [133]. Other approaches that have been considered for the improvement of PE systems include the paired pegRNA strategy [134], the use of engineered pegRNAs with 3′ extensions to minimize pegRNA degradation [135,136], or the manipulation of the cellular DNA repair pathway through the installation of strategic silent mutations in close proximity of the intended edit [137].

### 4.3. Allele Replacement

Many agronomic traits in higher plants are conferred by single-nucleotide substitutions, and subtle changes in gene structure that could affect the biochemical function of encoded product(s) and/or alter the levels of gene expression [138]. Indeed, single nucleotide polymorphisms (SNPs), transposon insertions, and other types of genetic modifications have been considered as the casual agents of mutations in more than half of the 62 analyzed domestication and diversification genes across 23 different crop species [139]. Moreover, it becomes increasingly apparent that the genetic complexity underlying domestication and crop-improvement-associated traits consists of a large number of minor-effect QTL alleles, likely organized in an epistatic network [140,141]. Thus, an improved versatility in the gene editing toolset, such as replacements and targeted insertions that could facilitate breeding by introducing novel alleles without linkage drag or generation of allelic variants that do not occur naturally [142], is necessary to address this complexity.

Creation of in planta targeted sequence variation can be achieved through gene targeting [143], utilizing the HDR-mediated pathway for the generation of pre-designed edits. This approach has been used to improve drought tolerance in maize by replacing the endogenous promoter of the ARGOS8 gene [144] and to improve the shelf-life of tomato by generating a precise replacement (T317A) in the ALC gene [145]. Nevertheless, the low rate (0.1–1%) of successfully recovered plants, due to the negligible occurrence of the HDR pathway in somatic cells, makes it rather impractical for routine allele replacement in crops, and hence its adoption in the fields of functional genomics and crop improvement is narrow. In contrast, the high-frequency production of indels has been demonstrated through the NHEJ-mediated repair pathway of targeted DSBs in rice, maize, *Arabidopsis*, and tomato [146,147,148,149]. This difference is a consequence of the suppression of HDR during the late S and G2/M phases of the cell cycle, as opposed to NHEJ, which operates regardless of the plant cells’ phase [150,151].

Several strategies that favor expression of the HDR pathway versus NHEJ repair have been reviewed recently [152]. Improving the availability of the donor repair template (DRT) by making it more accessible to the cell repair machinery and/or by increasing its copy number in the target cells has been tested in planta. Other approaches involve the positioning of DRT near the DSB site, resulting in an increase in the spatial availability of the DRT. Moreover, modified DRT architectures, such as the length and ratio of the homologous and non-homologous parts of the DRT, type and strandedness of the nucleic acid, presence of chemical modifications, and positioning of the DRT relative to the DSB, are also important elements for consideration in gene targeting experiments. Other approaches that have been considered are the utilization of alternative DSB inducers for increased efficiency of target cleavage and subsequently enhanced HDR, or the modulation of the DNA repair pathway to favor HDR by inactivating key genes promoting NHEJ [152].

## 5. Enabling Technologies

Although the CRISPR/Cas technologies described above provide tremendous promise towards the functional characterization of causal variants in crop plants, there are still bottlenecks in the gene editing pipeline that need to be addressed before high-throughput gene editing becomes routine. These challenges include improving guide RNA validation, developing an efficient in planta transformation system to bypass the time-consuming in vitro tissue culture and regeneration process, and finding more efficient ways to characterize mutations in the gene edited progeny.

### 5.1. CRISPR/Cas gRNA Validation and Confirmation of Editing Efficiencies

#### 5.1.1. In Vitro RNP Assay

Cas9 is an endonuclease that is targeted to a specific genomic region by a small single guide RNA (sgRNA). The cleavage efficiency of Cas9 varies greatly from one sgRNA to another sgRNA. The efficiency of the sgRNAs in performing the edits can be checked using a ribonucleoprotein (RNP) assay by in vitro digestion of target DNA with a mixture of purified Cas9 nuclease and synthetic sgRNAs. In practice, the target DNA segment can be amplified from genomic DNA using flanking PCR primers. This method is a simple, low-cost, and rapid method to identify efficient sgRNAs for desired genes in plants and other organisms [153]. This screening method allows a researcher to choose the best predicted sgRNAs designed from online sgRNA tools prior to deliver genome editing reagents into live plant cells [154]. The target DNA fragment, sgRNA, and Cas9 nuclease are combined and incubated for the in vitro cleavage reaction, followed by fragment analysis using gel electrophoresis to compare the cut versus uncut PCR products.

#### 5.1.2. Validation Using Protoplasts

**Figure 3:**
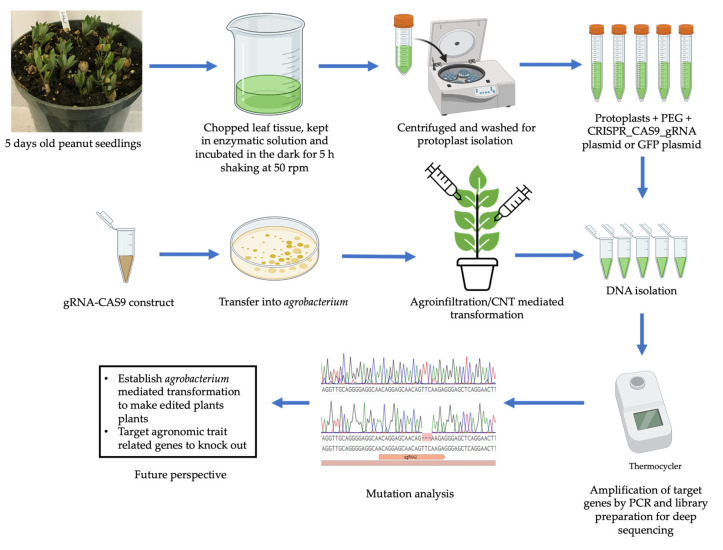
Overview of in vivo gene editing in plant protoplasts and using leaf infiltration (created with BioRender.com, accessed on 23 March 2022).

Plant “protoplasts” (whose name originates from the ancient Greek prōtóplastos, meaning “first-formed”) are whole plant cells without cell walls, and have been widely used in plant science research and crop breeding. Protoplast transformation/transfection involves the direct delivery of plasmid DNA to individual protoplasts using polyethylene glycol (PEG), electroporation or other methods [155]. Protoplast transformation has several benefits, including: delivery of multiple plasmids with high levels of cotransformation, no binary vector required, and providing the ability to check CRISPR construct efficiency in a short time. Isolation of high yield and good quality protoplasts depends on the proper starting tissue material and age of the plants [156,157]. For leguminous crops such as chickpea and soybean, fully expanded leaves are the best choice for protoplast isolation [158,159]. Temperature is another crucial factor for maintaining the viability of the isolated protoplasts: most plant protoplasts are stable at room temperature (23 °C–28 °C) [160]; however, cold storage is sometimes necessary. The optimum concentration of PEG and the duration of the PEG incubation time are other criteria that need to be considered for increasing transformation efficiency in protoplasts [160,161]. Protoplast transfection is commonly used in model organisms and crops to test the efficiency of gRNA design and Cas protein activity in vivo [161]. The genome editing reagents (DNA, mRNA, or RNPs) can be delivered into protoplasts via transfection; the plant protoplast platform is simple, robust, economical and generally applicable in multiple plant species to support the rapidly evolving advances and innovations of CRISPR technologies [153,157,161]. As generating stable genome-edited plants is complex and labor-intensive [161], it is necessary to evaluate the most effective Cas9-gRNA first. To evaluate the potential of the CRISPR-Cas9 system in planta, a reproducible system for the design, construction, and delivery of Cas9-gRNA needs to be developed and validated via in vitro and in vivo systems (Figure 3). For the in vivo assay, protoplast transformation can be used as a tool to express genes transiently as well as evaluate genome editing efficacy.

#### 5.1.3. Leaf Infiltration

Leaf infiltration is a simple and effective method for transient transfer of the gene of interest/recombinant DNA into host plant cells by physical or vacuum infiltration [162]. Agroinfiltration is the most common form of leaf infiltration, which leads to the transfer of single-stranded T-DNA from *Agrobacterium* to the plant cells [163]. Agroinfiltration can be achieved by different methods, such as syringe infiltration, vacuum infiltration, hydrogen peroxide-based agroinfiltration, leaf disc vacuum infiltration, spray-based agroinfiltration, and the detached leaf-based infiltration approach [164,165,166,167,168]. Agroinfiltration has been used with the CRISPR/Cas9 system for transient delivery of Cas9 and sgRNA for targeted gene mutations in plants [169]. This approach is widely performed for the determination of gRNA efficiency in genome editing constructs in vivo. The combinatorial approach of CRISPR-Cas editing and agroinfiltration has resulted in transformed tissues within 3 days and has been tested for CRISPR/Cas9-mediated genome editing targeting PDS gene in various plant species [170,171,172]. Most of the studies showed bleached patches in the infiltrated areas of leaves that can be examined through microscopic analysis to confirm the transient knockout of the PDS gene. Leaf infiltration, however, generally does not lead to stable transformation of germline cells and so will not be inherited in the next generation.

#### 5.1.4. Hairy Root Transformation with *Agrobacterium rhizogenes*

Another approach for gRNA validation is to use hairy root transformation with *Agrobacterium rhizogenes*, an approach that provides a faster method for transformation than *A. tumefaciens*, especially for legumes [173]. Transformation with *A. rhizogenes* also results in T-DNA transfer, but in this case resulting in dense, hairy roots containing the transgene. Hairy root transformation provides a rapid approach to test guide RNA (gRNA) efficiency in vivo and has been successfully used in soybean for CRISPR/Cas9 editing [174,175].

### 5.2. In Planta Transformation for High Throughput Editing

#### 5.2.1. *Agrobacterium tumefaciens*

In most cases, a CRISPR/Cas9 expression cassette, together with a selectable marker gene, is delivered to the plant cells through *Agrobacterium tumefaciens*- or biolistic delivery-mediated transformation methodologies. However, these methods generally require in vitro callus culture and regeneration processes that are lengthy, costly and labor-intensive. Furthermore, these conventional tissue culture-based transformation methods are genotype-specific and need to be re-optimized for each new genotype or cultivar, which significantly limits the application of genome editing to commercial varieties in major crops such as wheat (*Triticum aestivum* L.), maize (*Zea mays* L.), and soybean (*Glycine max* L.). In contrast, in planta methods take advantage of normal growth and reproduction and bypass the tissue culture processes, potentially offering a more efficient and widely applicable transformation system [176]. Floral dip in *Arabidopsis* has been widely used for transformation over many years, and more recently used to delivery of CRISPR/Cas9 components genome editing; however, it is generally limited to Brassicaceae species [177,178]. In a more recent in planta transformation study, meristematic tissues have been targeted for *Agrobacterium*-mediated delivery to demonstrate genome editing in tobacco and other species [179]. Although there have been limited successes, more optimization is needed to develop a robust in planta transformation system that will allow for rapid and efficient gene editing. 

#### 5.2.2. Carbon Nanotubes

Nanotechnology-based methods have already been established as inexpensive, easy, and robust techniques to transfer genes or other molecules into plant and animal cells with high efficiency and low toxicity [180,181,182]. Nanoparticles (NPs) are classified based on the base materials, such as carbon-based NPs, silicon-based NPs, metallic NPs, and polymer-based NPs; each of the particles transfer different loads into cells. For example, carbon nanotubes (CNTs) can transfer both RNA and DNA, but metallic NPs can only deliver DNA [183,184]. In addition, silicon-based NPs can carry DNA and proteins, and polymeric NPs (e.g., PEG) can transfer RNA, DNA, and proteins into cells [185,186,187]. Recently, efficient DNA delivery and strong protein expression were shown in *Nicotiana benthamiana* (tobacco), *Eruca sativa* (arugula), *Triticum aestivum* (wheat) and *Gossypium hirsutum* (cotton) leaves and arugula protoplasts using CNTs without transgene integration [188]. Moreover, gene silencing in plants has been achieved with no toxicity and no physiological disturbance after CNT-mediated transient gene expression in mature plants [189]. One of the most useful applications of CNTs will be to efficiently deliver plasmids coding for nuclease-based genome-editing proteins such as zinc finger nucleases, transcription activator-like effector nucleases, and Cas (CRISPR-associated) nucleases. Initial results in rice show that CNTs can be used to successfully deliver plasmid DNA into rice leaves and germinating seeds [190]. CNT-based delivery combined with an efficient in planta transformation protocol has the potential to provide high-throughput gene editing for functional validation and crop improvement.

#### 5.2.3. Developmental Regulators

Transformation of major cereal crops is difficult due to genotype recalcitrance and long and toilsome callus induction and regeneration protocols. Several developmental regulator genes have been characterized—for example, somatic embryogenesis receptor kinase (SERK4), LEAFY COTYLEDON1 (LEC1), LEAFY COTYLEDON2 (LEC2), BABY BOOM and WUSCHEL, which can enhance somatic embryogenesis during in vitro regeneration [191,192,193,194,195]. In particular, higher transformation efficiency was found in transformation-recalcitrant inbred maize lines, rice, wheat, sorghum and sugarcane after co-overexpression of both BBM and WUS2 genes [196,197]. However, overexpression of these genes also had some negative effects on plant phenotype [196]. There are also reports that ectopic expression of BBM and WUS2 using the promoter of maize phospholipid transferase protein (PLTP) and an auxin-inducible promoter increases transformation efficiency without having any aberrant phenotypes [198]. Moreover, overexpression of the wheat gene TaWOX5 dramatically improves the transformation frequency of wheat and five other cereal species and no negative effects on plant phenotype [199]. A similar approach has been used for gene-editing by de novo meristem induction through ectopic expression of developmental regulatory genes including BBM, WUS, SHOOT MERISTEMLESS (STM), and ISOPENTENYL TRANSFERASE (IPT) by *Agrobacterium* transformation [179]. This technology will be useful to produce gene-edited crops without a lengthy tissue culture process resulting in cost reductions and time savings.

### 5.3. Characterizing Gene Edited Plants

#### 5.3.1. Molecular Characterization

Several mutant screening methods have been developed, such as direct Sanger sequencing of PCR products, restriction enzyme (RE) site loss assay, T7 endonuclease I (T7EI) assays, polyacrylamide gel electrophoresis (PAGE)-based genotyping approach, high-resolution melting analysis (HRMA), and annealing at critical temperature PCR (ACT-PCR), ddPCR (digital droplet PCR), and high-throughput next generation sequencing based methods [200,201,202,203,204,205,206,207,208]. Among them, the polymerase chain reaction (PCR)-based assay, restriction enzyme (RE) site loss assay, and high-throughput next-generation sequencing are frequently used for characterization of target mutants. Another approach is to use capillary electrophoresis-based fragment analysis for high-throughput detection of insertion/deletions in gene-edited samples [209]. Traditional Sanger sequencing methods to identify homozygous mutants are time-consuming and laborious for large numbers of samples and may be unsuitable for heterogeneous samples [210]. However, targeted deep sequencing of a specific locus using next generation sequencing (NGS) is a feasible approach to identify not only both homozygous and heterozygous mutations but also the frequency of mutations of each target edit for genome-edited plants [211]. Moreover, this method is more cost effective and time efficient when running large numbers of samples at a time using a barcode adapter approach.

#### 5.3.2. Phenotypic Characterization

Genome editing by CRISPR has great potential in plant biology for unravelling gene function and improving agronomical traits, since CRISPR can be used to introduce allelic variants affecting phenotypes. Initially, CRISPR technology was used to generate loss-of-function mutant alleles with clear phenotypic effects. With the advancement of new CRISPR technologies such as base editing, prime editing, and dCas9 approaches, CRISPR now has the capacity to generate the gain-of-function alleles or allele variants at the genomic or epigenomic level [212]. Several studies have demonstrated the successful applications of base editors in a wide range of plants, including rice, maize, tomato, wheat, cotton and watermelon by gaining different characteristics such as herbicide resistance, improving grain size and yield, biotic stress tolerance, lipid metabolism, and nutritional improvement [170,174,213,214,215]. As more traits are targeted with gene editing, future applications can also leverage advances in precision phenotyping and machine learning to connect specific phenotypic differences across trait components with functional polymorphisms. 

## 6. Conclusions

The technology landscape supporting crop improvement efforts has undergone several large transformations over the past few decades. The initial rise of molecular markers led to large-scale mapping and selection of genes and QTLs controlling key traits for crop improvement. Advances in high-throughput genotyping techniques made genomic selection cost-effective, while high-throughput phenotyping techniques have enabled larger populations and more precise phenotypic characterization of breeding materials. At the same time, large-scale genomics initiatives, including whole genome sequencing, transcriptome profiling, and proteomics and metabolomics efforts, have provided an unprecedented amount of information on candidate genes and genetic pathways controlling traits of interest. Plant transformation advances led to transgenic crops for several traits but were bottlenecked by slow and laborious in vitro regeneration processes combined with an expensive deregulation system. Likewise, the emergence of CRISPR/Cas-based gene editing shows tremendous promise but is hampered by the lack of a high-throughput gene editing pipeline and limited knowledge of which functional nucleotide polymorphisms to target for maximum gains in crop improvement. As discussed in this review, these developments have now set the stage for a new phase in crop improvement that uses gene editing for functional allele validation to leverage the wealth of genetic resources for crop improvement. Enabling technologies will provide high-throughput gene editing for massively parallel selection at key causal variant sites, allowing molecular breeders to break out of the limitations of genetic recombination and usher in a new era of genetic gains and novel trait development for crop improvement to meet the challenges of the future.

## Data Availability

Not applicable.
